# Pre-emptive analgesia with methylprednisolone and gabapentin in total knee arthroplasty in the elderly

**DOI:** 10.1038/s41598-022-05423-4

**Published:** 2022-02-11

**Authors:** Henryk Liszka, Małgorzata Zając, Artur Gądek

**Affiliations:** 1 Department of Orthopedics and Physiotherapy, Jagiellonian University Medical College, Kraków, Poland; 2 Department of Orthopedics and Traumatology, University Hospital in Krakow, Jakubowskiego 2, 30-688 Kraków, Poland; 3 Department of Anatomy, Jagiellonian University Medical College, Kraków, Poland; 4 Department of Anaesthesiology and Intensive Care, Jagiellonian University Medical College, Kraków, Poland; 5 Department of Anaesthesiology, University Hospital in Krakow, Kraków, Poland

**Keywords:** Pain management, Geriatrics, Combination drug therapy, Reconstruction

## Abstract

The aim of this study is to assess whether administration of gabapentin and methylprednisolone as “pre-emptive analgesia” in a group of patients above 65 years of age would be effective in complex pain management therapy following total knee arthroplasty (TKA). One hundred seventy patients above 65 years were qualified for the study, with exclusion of 10 patients due to clinical circumstances. One hundred sixty patients were randomly double-blinded into two groups: the study group (80 patients) and the control group (80 patients). The study group received as “pre-emptive” analgesia a single dose of 300 mg oral (PO) gabapentin and 125 mg intravenous (IV) methylprednisolone, while the control received a placebo. All patients received opioid and non-opioid analgesic agents perioperatively calculated for 1 kg of total body weight. We measured (1) pain intensity level at rest (numerical rating scale, NRS), (2) life parameters, (3) levels of inflammatory markers (leukocytosis, C reactive protein CRP), and (4) all complications. Following administration of gabapentin and methylprednisolone as “pre-emptive” analgesia, the NRS score at rest was calculated at 6, 12 (p < 0.000001), 18 (p < 0.00004) and 24 (p = 0.005569) h postoperatively. Methylprednisolone with gabapentin significantly decreased the dose of parenteral opioid preparations (p = 0.000006). The duration time of analgesia was significantly longer in study group (p < 0.000001), with CRP values lower on all postoperative days (1, 2 days—p < 0.00001, 3 days—p = 0.00538), and leukocytosis on day 2 (p < 0.0086) and 3 (p < 0.00042). No infectious complications were observed in the first postoperative days; in the control group, one patient manifested transient ischemic attack (TIA). The use of gabapentin and methylprednisolone as a single dose decreased the level of postoperative pain on the day of surgery, the dose of opioid analgesic preparations, and the level of inflammatory parameters without infectious processes.

## Introduction

Total knee arthroplasty (TKA) is performed in older patients due to degenerative lesions and to improve the quality of life. The postoperative period is associated with inconveniences, including pain of maximal intensity on day 1^1^. Surgery, as a mechanical factor, initiates the pain process, incites nociceptors, evokes hyperalgesia, releases inflammatory mediators at the site of surgical incision and through damaging the vessels along with the anticoagulative and antiadhesive lining covering the vascular endothelial cells, initiates local as well as generalized inflammatory process^2–4^.

Prolonged administration of glucocorticoids in patients with osteoarthritis limits the inflammatory process and reduces chronic pain through inhibitory factors evoking hyperalgesia, synthesis of proinflammatory factors and blocking elements of the inborn immune system. The use of methylprednisolone at the dose of 125 mg as “pre-emptive” analgesia^5–10^ has been also initiated since 2013. The Lundbeck Foundation of Denmark has been recommending using the medication as an element of the Enhanced Recovery After Surgery (ERAS) protocol in TKA^5–9,11^.

Gabapentin is employed in managing chronic pain due to its’ effects exerted on cerebral modulation and perception of pain, as well as postoperative somnolence as an adverse effect, which is beneficial to the patient and fits well with multimodal pharmacotherapy of pain^12,13^.

### Objective

The objective of this study was to assess the employment of methylprednisolone and gabapentin in complex pain management during the perioperative period in patients above 65 years of age subjected to TKA. The authors attempted to answer whether administration of a single dose of methylprednisolone and gabapentin as “pre-emptive” analgesia would reduce: (1) the level of postoperative pain using the numerical rating scale (NRS) at rest and every 6 h starting on day 0, (2) the dose of parenteral analgesics agents, mainly opioids, used by the patients, (3) the occurrence of adverse effects that would delay early rehabilitation, (4) the inflammatory process parameters, and (5) maintain stable glycemic levels.

## Materials and methods

### Study design and enrollment of patients

This study was a single-center, prospective, double-blind randomized controlled trial conducted in a tertiary care hospital. The study started after obtaining approval at the Bioethics Committee Jagiellonian University meeting on the research plan presented. The Bioethics Committee of the Jagiellonian University, Kraków, Poland, confirmed its consent by letter no. 1072.6120.11.2020, 23/01/2020. The research was registered with ClinicalTrials.gov (ID: NCT04653415, 28/11/2020). All research was performed in accordance with relevant guidelines and the Declaration of Helsinki. After obtaining oral and written informed consent to participate in the study, consecutive patients above 65 years of age, were operated on with unilateral TKA in the period of June 1st to December 31st, 2019, and the ERAS protocols were recruited following the procedures. We excluded patients due to clinical circumstances that (1) restricted glucocorticoid administration, such as: diabetes type 1 and 2, CRP levels above normal values (≥ 5 mg/l), current chronic steroid treatment, peptic ulcers treated within the past 30 days, and (2) chronic pain such as: in the course of gonarthrosis, patients being treated in Pain Clinics, high intensity pain above 6 points on the NRS scale, inability to perform activities of daily living, and night pain at rest despite multimodal pharmacotherapy already requiring the use of additional transdermal and less often oral opioids.

### Randomization

Before surgery, all of the patients were randomized into two groups: the study (“pre-emptive analgesia” large dose of methylprednisolone 125 mg and gabapentin 300 mg) and the control (placebo 0.9% saline solution and tablet without pharmacological activity). The allocation was performed in the preanesthetic clinic by an individual not involved in the study, who prepared block randomization (block sizes 4 and 6), and used sealed envelope technique for all allocation concealments, including “pre-emptive analgesia” among the two sets. Each kit included a “pain reliever tablet” (gabapentin or placebo) and pre-filled syringe with “steroid” (methylprednisolone or saline solution) prepared by the Hospital Pharmacy. Clinical trial was coded by the topic manager without affecting the experimenters.

### Procedure

First and foremost, prior to anesthesia induction, each patient received prophylactically intravenous (IV) cephazolin 2.0 g as an antibiotic, tranexamic acid 1.0 g for hemostasis control, and an anti-emetic agent—ondansetron 8 mg. Patients were then subjected to the standardized procedure of subarachnoid anesthesia with subsequent unilateral femoral nerve block on the operated side, followed by the surgical procedure—unilateral TKA (P.F.C. SIGMA DePuy Synthes) using parapatellar approach. Fluid crystalloids administration was standardized to 12 ml/kg in the first hour of surgery, followed by 6 ml/kg for the remainder of surgery. Packed red blood cells were prepared in case of blood loss exceeding 600 ml and hemoglobin concentration reaching < 10 g/l during the time of operation. Pain management was carried out based on the results of the NRS scale at rest, as well as assessment of the duration of the analgesic femoral nerve block. The time of the femoral nerve blockade separated two points on the NRS scale and were determined by return of the superficial sensation of skin temperature changes (cold vs warm). The measurements were carried out every hour, and statistical calculations were performed every 6 h. On postoperative day zero, analgesia was started if the pain level on the NRS scores was 2–4 points, with intravenous (IV) paracetamol one to four times per day up to a maximum dose of 4 g, or metamizole one to two times a day up to a maximum dose of 5 g. When the pain level was at an NRS score of ≥ 4, the patients received intravenous (IV) oxycodone hydrochloride as PCA (patient-controlled analgesia). All pain medications were calculated for 1 kg of total body weight. In keeping with the ERAS protocol, on the day of surgery, patients received oral fluids and meals, and were mobilized and rehabilitated. On postoperative day zero, rehabilitation included standing up, walking up to 20 m with crutches and continuous passive movement with a rehabilitation splint. On the next day, walking distance with crutches was increased, with addition of stair exercises and continuation of passive movements with the rehabilitation splint in order to obtain a knee flexion of 90 degrees. Each patient had wound drainage applied by the surgeon during the operation. Fluid samples from the drains were collected to assess the level of CRP. The wound drainage was removed 24 h postoperatively.

In the preoperative room, patients received “pre-emptive analgesia” as one of the prepared coded kits. The study Group M received a kit containing oral (PO) gabapentin 300 mg and a pre-filled syringe of intravenous (IV) methylprednisolone 125 mg. The control Group K received an oral (PO) placebo and a pre-filled syringe of intravenous (IV) 0.9% saline solution. The statistical analysis of the groups addressed the demographic dates, life parameters, general condition in keeping with the ASA (American Society of Anesthesiology), POSSUM (Physiologic and Operative Severity Score for the enUmeration of Mortality and Morbidity) score, total dose of analgesic medications administered parenterally calculated for 1 kg of total body mass in response to values of NRS at rest on day 0, time of administration of the first dose, and duration of peripheral nerve block. On the day of surgery and on subsequent days, indicators were made based on glycemic levels and inflammatory markers: C-reactive protein (CRP) and leukocytosis (WBC) levels. The primary outcome was pain intensity level measured by the NRS scale. The secondary outcomes were demographic dates, life parameters, general condition in keeping with the ASA, POSSUM Mortality and Morbidity score, total dose of analgesic medications, time of administration of the first dose, duration of peripheral nerve block, glycemic levels and inflammatory markers levels: C-reactive protein (CRP) and leukocytosis (WBC).

### Statistical analysis

The statistical analysis was performed using the T-Student test for independent groups employing the Cochran-Cox modification. A two-way ANOVA test or linear correlation (r Pearson) was used to determine the influence of two different categorical independent variables on one continuous dependent variable (NRS pain score); the resultant statistical significance was p < 0.05 (PQStat v 1.6.8 software). A priori statistical power analysis was also conducted to validate the adequacy of the sample size. An NRS pain score was used to calculate the sample size. A sample size of 73 in each group was calculated; the level of significance was fixed at 5% and the power of the study at 80%. Expecting a 10% drop out rate, sample size was calculated at 80 for each group (total = 160).

## Results

**Figure 1:**
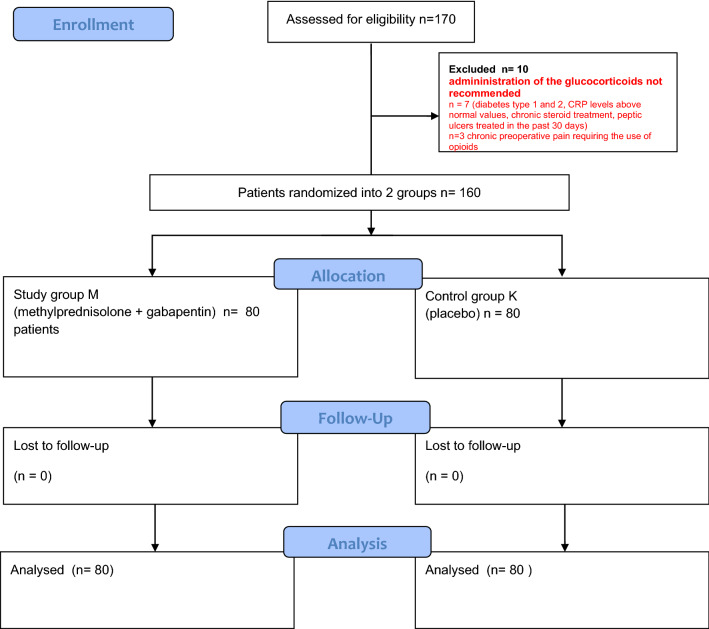
A schematic diagram showing the CONSORT flowchart. Patient selection and randomization methylprednisolone + gabapentin vs placebo as *pre-emptive analgesia* TKA (total knee arthroplasty).

The analysis included a group of electively picked 170 patients above 65 years of age who had a unilateral TKA operation performed in the period of June 1st to December 31st, 2019, with the procedures following the ERAS protocols in our surgical department. From the study, 10 patients were excluded due to clinical circumstances that (1) restricted glucocorticoid administration, such as: diabetes type 1 and 2, CRP levels above normal values (≥ 5 mg/l), current chronic steroid treatment, peptic ulcers treated within the past 30 days, and (2) chronic pain such as: in the course of gonarthrosis, patients being treated in Pain Clinics, high intensity pain above 6 points on the NRS scale, inability to perform activities of daily living, and night pain at rest despite multimodal pharmacotherapy already requiring the use of additional transdermal and less often oral opioids. The schematic flowchart as per the CONSORT is given in Fig. 1.

**Figure 2:**
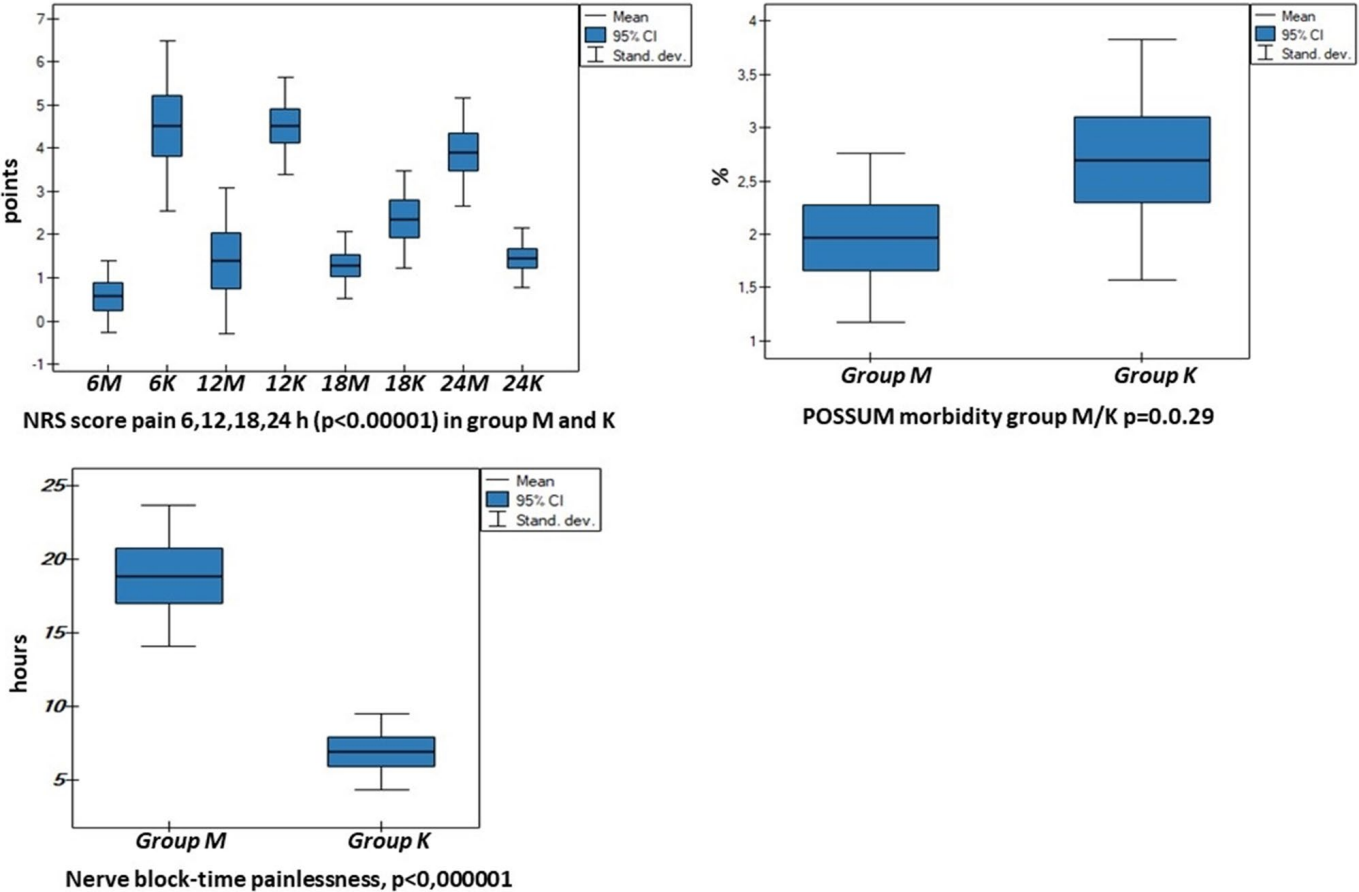
Correlation parameters between group M and K: NRS score pain 6, 12, 18 h (p < 0.00001), POSSUM morbidity (p = 0.029), nerve block—time painlessness (p < 0.000001).

The two groups of patients were numerically comparable, consisting of 80 patients in each group. No differences were seen between the groups in mean age, duration of surgery, initial and postoperative life parameters (excluding the pulse rate on day 0 in the control group), perioperative risk according to the ASA scale, postoperative efficiency, possible complication development, perioperative mortality on the POSSUM scale, as well as comorbidities. Significantly longer time of postoperative hospitalization values were seen in the control group (Tables 1, 2). This finding confirms the statistical significance in the POSSUM morbidity difference (p < 0.029) between the groups, the difference being higher in the control group (Table 2, Fig. 2).

**Table 1:** General data describing the analyzed elderly patients (over 65 years old) divided into the study group (M) and the controls (K).

Data	Group (M)	Group (K)	p-value
n	80	80	
Age (years)	73.5 ± 7.47	71.36 ± 5.23	0.21
Mean hospitalization time (days)	3.98 ± 0.92	5.81 ± 1.45	0.010
Mean postoperative hospitalization time (days)	3.01 ± 0.96	4.87 ± 1.15	0.0113
Postoperative MAP (mmHg)	96.39 ± 8.23	98.87 ± 10.99	0.328
Postoperative pulse (x/min)	68.75 ± 15.08	77.78 ± 21.50	0.0093
Postoperative Sp02 (%)	98.17 ± 0.98	98.60 ± 0.70	0.053
Wound drainage (ml)	353.57 ± 126.14	325.75 ± 105.41	0.351

*MAP* mean arterial pressure.

**Table 2:** Distribution of comorbidities and anesthesia in the analyzed patients aged above 65 years old divided into the study group (M) and the controls (K).

Data	Group M		Group K		p-value
	n	%	n	%	
n	80	100.00	80	100.00	
Hypertension arterial	80	100.00	80	100.00	
Atrial fibrillation, arrhytmia supraventricular	9	11.25	9	11.25	
IHD, myocardial infarction in anamnesis	8	10	9	11.25	0.564
COPD grade III	6	7.5	5	6.25	0.789
TIA in anamnesis	6	7.5	5	6.25	0.621
Cerebral tumor in anamnesis	1	1.25	2	2.5	0.356
Regional anesthesia	80	100.00	80	100.00	
Femoral nerve block-analgesia	80	100.00	80	100.00	
ASA II	66	82.5	60	75	0.326
ASA III	14	17.5	20	25	0.129
POSSUM physiological (points)	17.42 ± 3.28		18.06 ± 4.45		0.537
POSSUM morbidity (%)	26.22 ± 12.91		26.54 ± 15.13		0.029
POSSUM mortality (%)	4.38 ± 3.55		4.94 ± 3.16		0.513

*IHD* ischaemic heart disease, *COPD* chronic obstructive pulmonary disease, *TIA* transient ischaemic attack, *ASA* American Society of Anesthesiology Score, *POSSUM* physiological and operative severity score for the enumeration of morbidity and mortality.

In the postoperative period the analysis focused on pain sensation on day 0 based on the NRS scale at rest calculated every 6 h, and the dosage of analgesic agents administered on the day of surgery. The duration of femoral nerve block and the elapsed time without pain before NRS pain score reached a level of 2 points correlates with the time of administration of the first postoperative analgesic dose (Fig. 2, 4, 5). These results were significantly longer in the study group, indicating that the “pre-emptive” analgesia in the study group helped with pain control in the postoperative period.

**Figure 3:**
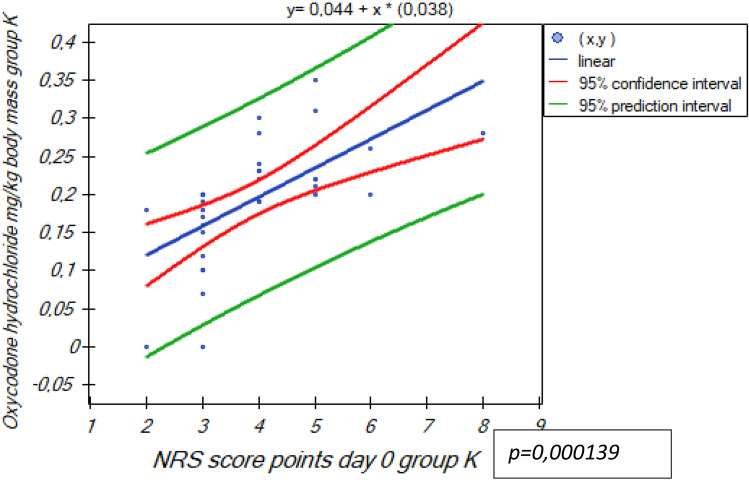
Linear correlation r-Pearson NRS points day 0 and oxycodone hydrochloride mg/kg body mass group K.

Only 21 of 80 subjects (26%) received oxycodone hydrochloride in view of an NRS score at rest ≥ 4 in the study group, whilst 71 of 80 patients (89%) in the control group. A statistically significant difference (p = 0.000139) is confirmed with a positive linear correlation (r Pearson) Fig. 3.

**Figure 4:**
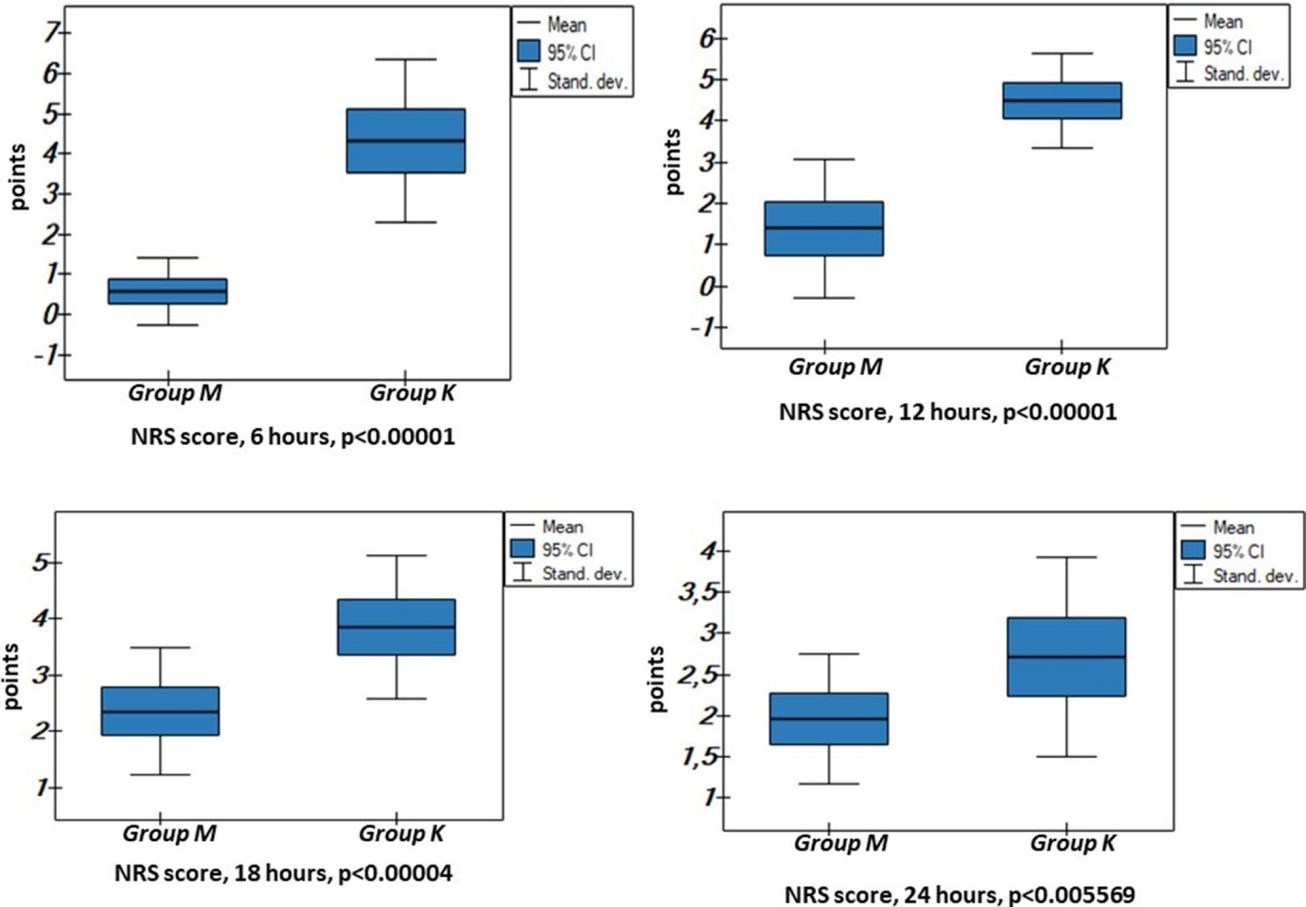
NRS level (points) at rest at time points at 6, 12, 18 and 24 h postoperatively in the analysed patients above 65 years of age divided into the study (M) and control (K) groups.

The analysis in the postoperative period also included triggers of pain such as inflammatory parameters in the two groups: leukocytosis (WBC) levels and CRP values on postoperative day 0, 1, 2, 3 in blood and fluid collected from surgical wound drains. Glycemic levels in consecutive postoperative days were determined (Table 3, Figs. 2, 3, 4).

**Table 3:** Leukocytosis CRP and glucose levels in the analyzed patients aged above 65 years old divided into the study (M) and control (K) groups.

Data	Group (M)	Group (K)	OR	− 95 CI	p-value
n	80	80			
Initial blood leukocytosis level (× 10^3^/µl)	6.82 ± 1.38	6.91 ± 1.88	− 0.083	− 0.94/0.77	0.846
Initial blood leukocytosis level—day 0 (× 10^3^/µl)	11.63 ± 2.52	10.92 ± 2.50	0.714	− 0.57/2.00	0.272
Blood leukocytosis level—day 1 (× 10^3^/µl)	13.78 ± 3.53	11.83 ± 3.35	1.94	0.17/3.71	0.031
Blood leukocytosis level—day 2 (× 10^3^/µl)	10.08 ± 2.48	11.96 ± 2.85	− 1.88	− 3.26/− 0.49	0.0086
Blood leukocytosis level—day 3 (× 10^3^/µl)	8.24 ± 1.85	10.96 ± 2.93	− 2.71	− 4.16/− 1.27	0.00042
Initial blood CRP level (mg/l)	3.09 ± 3.33	2.69 ± 1.55	0.39	− 0.90/1.69	0.543
Blood CRP level—day 0 (mg/l)	8.91 ± 12.07	18.33 ± 12.69	− 9.42	− 15.8/− 3.03	0.0045
Blood CRP level—day 1 (mg/l)	28.39 ± 21.57	61.06 ± 24.57	− 32.66	− 44.61/− 20.71	< 0.000001
Blood CRP level—day 2 (mg/l)	53.92 ± 41.89	105.52 ± 32.68	− 51.60	− 70.71/− 32.48	< 0.000001
Blood CRP level—day 3 (mg/l)	53.85 ± 51.04	97.12 ± 36.12	− 43.27	− 66.83/− 19.72	0.000538
Wound drainage fluid CRP level (mg/l)	8.09 ± 10.61	5.29 ± 4.37	2.79	− 1.24/6.84	0.171
Initial blood glucose level (mmol/l)	6.13 ± 1.13	5.79 ± 1.10	0.33	− 0.23/0.91	0.247
Blood glucose level—day 0 (mmol/l)	7.63 ± 0.90	7.66 ± 1.05	− 0.02	− 0.53/0.48	0.910
Blood glucose level—day 1 (mmol/l)	7.70 ± 0.83	7.94 ± 0.90	− 0.23	− 0.68/0.21	0.295
Blood glucose level—day 2 (mmol/l)	6.82 ± 0.62	7.14 ± 0.69	− 0.31	− 0.66/0.02	0.068
Blood glucose level—day 3 (mmol/l)	5.79 ± 0.58	5.88 ± 0.53	− 0.09	− 0.38/0.19	0.505

*CRP* C reactive protein, *OR* odds ratio, *CI* confidence interval.

## Discussion

The large joint arthroplasty in agreement with the ERAS protocol requires analgesic management for initiation of rehabilitation as early as on the day of surgery^14,15^. This preceding mobilization limits the time of hospitalization, as well as the risk of infectious and other complications^1,2,14,15^.

**Figure 5:**
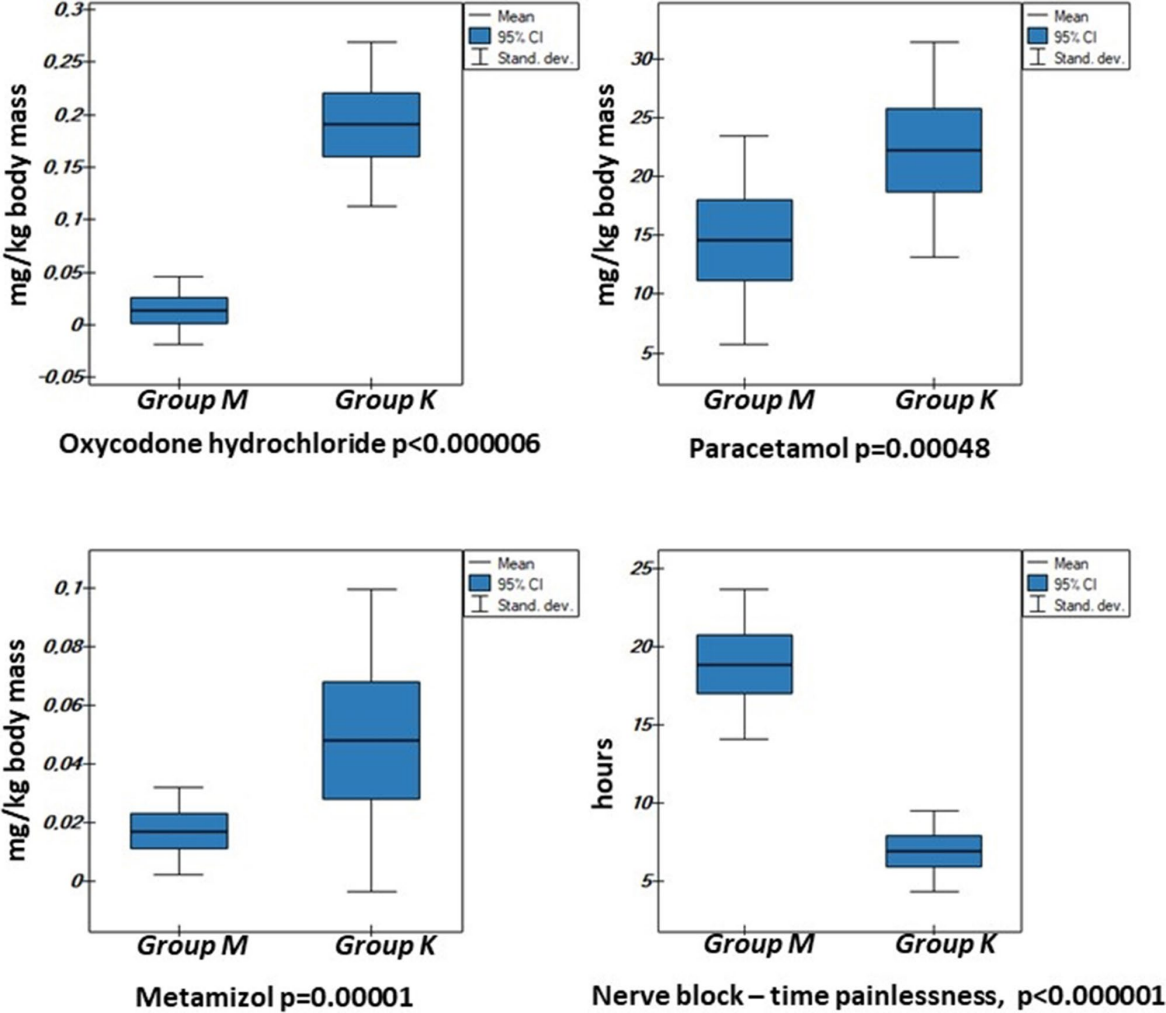
Dose of oxycodone hydrochloride in mg/kg body weight, paracetamol in mg/kg body weight, metamizole in g/kg body weight and time of postoperative analgesia in hours (h) not requiring administration of an analgesic agent in the analyzed patients above 65 years old divided into the study (M) and control (K) groups on day 0.

The mean postoperative hospitalization in the present material did not differ from data currently reported in publications and meta-analyses^7,11,15–18^ equaling 3.01 days in the study group. In the control group this time was significantly longer by 2 days (p = 0.0113) due to (1) prolonged and more severe pain on day 0, as illustrated by the level of pain on the NRS scale, which necessitated employing higher doses of analgesics including opioids (Figs. 3, 4, 5), (2) difficulties in achieving flexion movement of the knee up to 90 degrees was noted in two patients whose hospitalization was prolonged until days 8 and 9, and (3) development of significant unexpected complications to the overall health of two patients in the control group according to the ASA score (compensated hypertension in medical history): an 82-year old male with transient ischemic attack (TIA) on post-op day 2, discharged on post-op day 20 without any focal signs, and a 78-year old female with an episode of atrial fibrillation on post-op day 2, without causing a significant delay to discharge in comparison to the control group. POSSUM morbidity score shows these unexpected complications from the control group (Table 2, Fig. 2).

The ERAS protocol reduced the postoperative time of hospitalization following TKA an average of 3 days, similar to our study. The observation is illustrated by the meta-analysis addressing the years 1990–2015 presented by Osinski et al.^16^. Similarly, in Gotland’s hospitals in the period of 2011–2015 the postoperative hospitalization time of 6374 patients following TKA was shortened from 5 to 3 days (patients classified ASA I and II score, irrespectively of age)^17^. Vehmeijer et al. presented the evaluation of the so-called “outpatient” hospitalization after TKA based on data from Danish centers in the years 2014–2017, only 15 to 20% of patients were discharged earlier than 23 h of postoperative hospitalization, the remaining individuals required hospitalization longer than 1 day^18,19^.

The analysis of 1246 patients by Osinski et al. evaluated the effect of the type of anesthesia with analgesic peripheral nerve block employed in the course of unilateral TKA. The mean postoperative hospitalization time was shorter by 0.9 day in patients who received regional anesthesia as compared to general anesthesia and were able to achieve better knee flexion by a mean value of 10 degrees by postoperative day 4^16,20^. In our procedures, all patients received regional anesthesia with analgesic peripheral nerve block. In the study and control groups, we observed two patients with difficulties in achieving satisfactory flexion of the operated joint. Two patients in the study group achieved satisfactory flexion of the joint in half the time by post-op day 5, compared to the control group’s average of post-op day 8 and 9. This difference in time resulted from the studied variable: administration of IV methylprednisolone and PO gabapentin as “pre-emptive analgesia” as part of a complete pharmacological sequence of “multimodal pain therapy”.

Gabapentin, an anti-epileptic drug, shows structural similarity to an inhibitory neurotransmitter γ-aminobutyric acid (GABA) in the central nervous system, binding to the subunit α-2-δ G proteins which regulate voltage gated calcium channels (VGCC), and inhibit excitability and hypersensitivity of neurons of the spinal cord and central nervous system. Gabapentin also reduces activity of the NMDA spinal cord receptors. It causes reduction of pain and hyperalgesia^12,13^. Gabapentin used as an analgesic medication due to its inhibitory effects exerted on the nociceptors, is employed alone or in combination with celecoxib as “pre-emptive” analgesia^15^. A significant decrease of the dose of analgesic preparations, including opioid agents, as employed in pain management on the day of TKA followed by their use in consecutive three days 1 h before mobilization allowed for effective rehabilitation and achieving proper degree of knee joint flexion as presented by Lindberg-Larsen et al.^8^. Gabapentin has no effect on the level of blood morphotic elements, including leukocytosis, glycemia and CRP.

Glucocorticoids, including synthetics such as dexamethasone and methylprednisolone, maintain the integrity of the blood vessel lining that directly covers the endothelial cells, which in turn preserve anticoagulative and antiadhesive properties. This has an antiedematous effect and restricts the systemic initiation of inflammation. Every surgical procedure, including arthroplasties, breaks the continuity of the vascular endothelium, increasing vascular permeability, leading to edema formation and initiation of the inflammatory cascade in the operative site. Through effects on the activity of cyclooxygenase and lipoxygenase, methylprednisolone imposes restrictions on factors that stimulate nociceptors and directly affects the neuronal pathway of acute pain. Meta-analyses based on studies employing methylprednisolone and placebo present a significant difference in pain assessment using the VAS/NRS scale after 6, 12, 24 and 48 h, and in narcotic agents (morphine preparations) administered parenterally per 1 kg of total body mass on the day of surgery^2–5,7–11,14^ as an element of multimodal analgesia in orthopedic surgery^10,21–24^.

The authors analyzed 170 patients above 65 years of age. In some populations this is still the age considered as the “older patient” group. More frequently in literature, the author defines the elderly group as patients being above 85 years of age, since this is the age at which a progressive decline of the functional reserve occurs, leading to increased morbidity and mortality. At present, it is difficult to follow the opinion of biologists, related to the progress in life and in medical sciences to include patients whose age starts at 65 or even 75 years of life to be included in the group of “elderly patients”^25^. Our materials showed no intergroup differences in the initial state, however, severity of comorbidities with respect to their state was measured by the ASA and POSSUM scales (Table 2). First complication was a TIA which occurred in an 82-year old male from the control group who had no prior abnormalities with the blood flow to the central nervous system (CNS) as preoperatively screened with an echocardiograph, electrocardiograph, and imaging of the cervical and spinal vessels. Various causes of the complication were explored since the problem occurred in the control group, which did not receive methylprednisolone. This agent evens out the circulatory parameters by stabilizing the vascular endothelial lining with antiadhesive and anticoagulant properties during the postoperative period^4,9^. The second complication was an atrial fibrillation in a 78-year old female without any cardiac dysrhythmias in anamnesis, and a previous 24-h Holter monitoring procedure. With exception of the above, the present authors did not observe any other systemic complications or deaths.

The staff of the Orthopedic Center, University Hospital of Copenhagen, did not observe any significant difference of circulatory system parameters on the day of surgery in the course of mobilization and rehabilitation in TKA patients from two groups, one with “pre-emptive” analgesia with methylprednisolone and the control with placebo administration^21^. Study patients did not demonstrate any signs of subjective intolerance of verticalization as opposed to the control group where 40% of the individuals interrupted verticalization^21^. In our study, the authors did not observe a statistically significant difference in the mean arterial pressure on postoperative day 0 between groups, despite rehabilitation and mobilization (Table 1).

In our study group, methylprednisolone with gabapentin as “pre-emptive” analgesia significantly decreased the employed dose of all parenteral analgesics: oxycodone hydrochloride, paracetamol and metamizol in mg/kg body weight per day. The time of the first opioid dose administration after surgery measured in hours differed between the two groups (Fig. 5). This was the consequence of pain experienced at rest as measured by the NRS scale every hour, and statistically calculated every 6 h postoperatively (Fig. 4). This explains the significantly higher pulse in the postoperative period in the control group as compared to the study group (Table 1). Starting in 2007, publications have been presenting the effects of preoperative administration of dexamethasone and subsequently methylprednisolone on postoperative pain, hospitalization time, infectious complications, stability of the circulatory system following initial mobilization, as well as pain at rest and during activity^6,7,15,17–19,21,23,24,26–29^.

Degenerative lesions involving the peripheral neurons occurring in the process of aging lead to prolonged conduction within all peripheral nerves. The excitability threshold of sensory receptors is elevated, which is also true for pain receptors^25^. The peripheral nerve block time, measured as the time of subarachnoid anesthesia, is prolonged in the elderly. In this study, the time of subarachnoid neuraxial anesthesia with supplementary peripheral blockade of the femoral nerve was significantly different in the two groups, being almost threefold longer in the study group as compared to the control. As the material encompassed patients above 65 years of age whose reactions to topical anesthesia medications were similar, the difference in time resulted from one variable: administration of methylprednisolone and gabapentin in “pre-emptive” analgesia (Fig. 5).

The literature on the subject evaluated parameters such as CRP level, endogenous anti-inflammatory protein pentraxin-3, and markers of vascular endothelial dysfunction that initiate inflammatory processes: syndecan-1, thrombomodulin, sE-Selectin, and vascular endothelial growth factor (VEGF)^4,8,9,24,26–28^. In 2017, Lindberg-Larsen with coworkers demonstrated significantly higher levels of pentraxin-3 in groups of patients who were administered a single dose of methylprednisolone within the first 24 h postoperatively, and documented the absence of topical and generalized inflammatory processes^22^. Numerous authors demonstrated the CRP level lacking significance when the two groups were compared^24^. In our present study, analysis of the following inflammatory parameters included: significantly higher leukocytosis on postoperative day 1 in the study group as opposed to the control, yet on subsequent days (day 2 and 3 postoperatively), the leukocytosis levels were significantly lower (Table 3). In turn, CRP values were significantly lower throughout all postoperative days in the methylprednisolone and gabapentin administered patients as compared to the control group. CRP levels obtained from the drainage fluid did not differ in the two groups. The two groups were initially statistically comparable with respect to leukocytosis and blood CRP values (Table 3). Follow up results in the study group at 1 month and 1 year on the occurrence of inflammatory complications are prepared for publication.

As it was demonstrated in the present material, a single dose of methylprednisolone and gabapentin did not significantly affect fluctuations of glycemic levels in the analyzed patients; however, it should be emphasized that patients with glucose intolerance were excluded from the study (Table 3). Similar observations are reflected in data reported in the literature on the subject: among others, by Ghironzi et al.^25^.

Our study has some limitations that should be taken into account. First, we assessed selected laboratory parameters that were considered important. Second, we used subjective NRS scores for assessment of pain intensity level, as it is commonly accepted and used.

PROSPECT (procedure specific postoperative pain management) Working Group and the European Society of Regional Anaesthesia and Pain Therapy formulates guidelines for pain therapy with numerous publications outlining the treatment of acute pain in selected major operations. Multimodal analgesics with numerous agents and adjuvants are introduced while employing nerve blocks in order to eliminate narcotic substances and their adverse effects. Multimodal analgesia after knee joint replacement is pending current guidelines. Application of gabapentin is controversial as shown by Kang et al. in a meta-analysis based on 7 studies that compared administration of gabapentin versus placebo. Gabapentin did not demonstrate a decrease in postoperative pain and morphine consumption^30^.

In our study, gabapentin as a “pre-emptive analgesic” in combination with methylprednisolone showed signs of positive effect in reduction of pain and opioid doses after TKA on postoperative day zero.

## Conclusion

In summary, the use of a single dose of gabapentin and methylprednisolone as “pre-emptive analgesia” in the study group of patients above 65 years old (1) measurably decreased postoperative pain levels as assessed by the NRS scale at rest during all time intervals on the day of the unilateral TKA surgery, and decreased the dose of analgesic agents, including opioid preparations (oxycodone hydrochloride), (2) significantly lowered the levels of inflammatory parameters: CRP values on all postoperative days and leukocytosis (WBC) levels on postoperative day 2 and 3, (3) showed support of circulatory system stability, (4) demonstrated no statistical difference in glycemic levels, and (5) prevented any adverse effects that would delay early rehabilitation in accordance with the ERAS protocol.

## Data Availability

Data will be made available on request.
